# Does moxibustion work? An overview of systematic reviews

**DOI:** 10.1186/1756-0500-3-284

**Published:** 2010-11-05

**Authors:** Myeong Soo Lee, Jung Won Kang, Edzard Ernst

**Affiliations:** 1 Policy Division, Korea Institute of Oriental Medicine, Daejeon, South Korea; 2 Division of Standard Research, Korea Institute of Oriental Medicine, Daejeon, South Korea; 3 Complementary Medicine, Peninsula Medical School, University of Exeter, Exeter, UK

## Abstract

**Background:**

Several systematic reviews (SRs) have assessed the effectiveness of moxibustion for a range of conditions, often with contradictory conclusions. Our aim was to provide a critical evaluation and summary of these data.

**Methods:**

Electronic searches were conducted to locate all SRs of moxibustion for any condition. Data were extracted by two authors according to predefined criteria.

**Results:**

Ten SRs met our inclusion criteria, which related to the following conditions: cancer, ulcerative colitis, stroke rehabilitation, constipation, hypertension, pain conditions and breech presentation. Their conclusions were contradictory in several instances. Relatively clear evidence emerged to suggest that moxibustion is effective for breech presentation.

**Conclusions:**

Based on evidence from the currently available SRs, the effectiveness of moxibustion has been demonstrated for several conditions; however, due to the poor quality of the primary studies, there remains considerable uncertainty.

## Background

Moxibustion is an East Asian therapeutic method that uses the heat generated by burning herbal preparations containing *Artemisia vulgaris *to stimulate acupuncture points [1]. According to the theory of traditional medicine, heat is usually applied to acupuncture points during moxibustion to cure diseases by regulating the function of meridians and visceral organs. A possible explanation for how moxibustion works is that the heat stimulates acupuncture points, which increases *qi *circulation and relieves *qi *stagnation, leading to an improved disease state [2].

Acupuncture stimulation, which involves thrusting or twisting needles, results in various biochemical reactions that can have effects throughout the body. Unlike acupuncture, moxibustion uses heat stimulation at various temperature levels, ranging from mild skin warming to tissue damage from burning. This heat stimulation can yield inflammatory responses and induce vascular changes [2].

Although moxibustion is often used as a symptomatic treatment for a wide range of conditions in clinical practice, e.g., arthritis, gastrointestinal problems, gynaecological complaints and stroke rehabilitation, its clinical effectiveness remains uncertain [3-5], and many experts doubt its biological plausibility. Numerous clinical trials have emerged; however, their results are contradictory. Thus, SRs assessing the summary of this evidence may bring clarification. To date, several such articles have been published. Unfortunately, however, the conclusions drawn in these publications are also conflicting.

This overview is aimed at summarising and critically evaluating all SRs on moxibustion as a symptomatic treatment for any condition. Our ultimate goal is to provide clinicians with clearer guidance regarding the value of this therapy.

## Methods

The following databases were searched on July 22, 2010 without language restrictions: Medline, EMBASE, AMED, CINHAL, the Cochrane Library, six Korean Medical Databases (Korean Studies Information, DBPIA, Korea Institute of Science and Technology Information, Korea Education and Research Information Service, KoreaMed and Korean National Assembly Library) and Chinese Databases (CNKI). In addition, our extensive departmental files were searched by hand. The keywords used in the search were (systematic review OR meta-analysis) AND (moxa OR moxibustion). Articles were included if they related to a formal SR or meta-analysis on any type of moxibustion as a treatment for any type of condition. Reviews, comments and overviews without a systematic methods section were excluded.

To be included, the SR had to be concerned specifically with the effectiveness of moxibustion and include evidence from at least two controlled clinical trials. SRs evaluating moxibustion together with acupuncture without separate evaluation of each approach were excluded.

Key data were extracted independently by two authors (MSL & JWK) according to predefined criteria, including conditions, number of primary studies, methodological quality of the primary studies, conclusion of each SR, and data related with searching. Disagreements were resolved by discussion between the authors. Judgement about the quality of the primary studies was adopted from the respective SRs. The Overview Quality Assessment Questionnaire (OQAQ) was used to evaluate the methodological quality of all included SRs [6]. In the OQAQ, the score ranges from 1 to 7. A score of 3 or less was considered as indicative of major flaws, whereas a score of 5 or more suggested only minor flaws. The two authors did these assessments independently, and discrepancies were settled by discussion.

## Results

**Figure 1:**
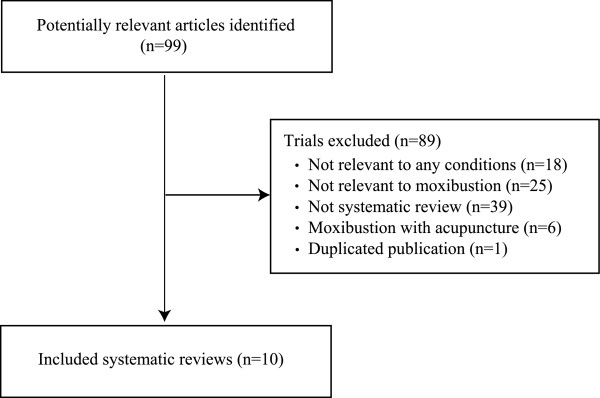
**Flow chart of publication selection process**.

Our searches generated 99 hits, and 10 articles met our inclusion criteria (Figure 1, Table 1) [3,7-15]. These studies included a wide range of conditions, including cancer [11], ulcerative colitis [10], stroke rehabilitation [12], several pain conditions [13], constipation [14], hypertension [15] and breech presentation [7-9]. Approximately half of the SRs arrived at a positive conclusion [8,9,11-13]. Most of the SRs were of good quality, but all had to rely on poor quality primary studies.

**Table 1:** Systematic reviews on moxibustion for health care

First author Year (Ref)	Condition	No. of primary studies	Methodological quality of primary studies^†^	Quality of SR OQAQ*	Meta-analysis	Conclusion (quote)^†^	Authors' result^†^
Kim (2010) [3]	Various conditions	48 RCTs	Poor	5	Ulcerative colitis 1) Moxa vs. drug (2 RCTs), RR, 2.20, 95% CIs,1.37 to 3.52, P = 0.001, I^2 ^= 0% 2) Moxa vs. wait control (2 RCTs), RR, 1.19, 95CIs, 0.88 to 1.60, P = 0.26, I^2 ^= 40% 3) Moxa plus postual care vs. postual care, RR, 1.51, 95%CIs, 1.10 to 2.08, P = 0.01, I^2 ^= 86%	Our results did not support the effectiveness of moxibustion in specific disease	**+/-**

Coyle (2005) [7]	Breech presentation	3 RCTs	Poor	7	None	There is insufficient evidence to support ...	**+/-**

Li (2009) [8]	Breech presentation	17(10 RCTs and 7 CCTs)	Poor	7	1) Moxa vs. no treatment (3 RCTs), RR, 135, 95%CIs, 1.20 to 1.51, P < 0.00001 2) Moxa vs. knee-chest position (3 RCTs), RR, 1.30, 95%CIs, 0.95 to 1.79, P = 0.1 3) Moxa plus other treatment vs. other treatment (2 RCTs), RR, 1.36, 95%CIs, 1.21 to 1.54, P < 0.00001	...tend to be effective...	+

Vas (2009) [9]	Breech presentation	7 RCTs	Variable	7	Moxa vs. control (6 studies), RR, 1.36, 95% CIs, 1.17 to 1.58, P < 0.0001	Moxibustion at ...BL67 has been shown to produce a positive effect...	+

Lee (2010) [10]	Ulcerative colitis	5 RCTs	Poor	7	Moxa vs. drug (5 RCTs), RR, 1.24, 95% CIs, 1.11 to 1.38, P < 0.0001, I^2 ^= 16%	Current evidence is insufficient ...	**+/-**

Lee (2010) [11]	Cancer	5 RCTs	Poor	6	Moxa plus chemotherapy vs. chemotherapy 1) Response rate (4 RCTs), RR, 1.04, 95% CIs, 0.94 to 1.15, P = 0.43, I^2 ^= 26% 2) Side effects (2 RCTs), RR, 0.38, 95% CIs, 0.22 to 0.65, P = 0.0005, I^2 ^= 0%	The evidence is limited to suggest moxibustion is an effective supportive cancer care in nausea and vomiting.	+

Lee (2010) [12]	Stroke rehabilitation	9 RCTs	Poor	5	Moxa vs. standard care 1) Motor function (3RCTs), SMD, 0.72, 95% CIs, 0.37 to 1.08, P < 0.0001, I^2 ^= 5% 2) Activities of daily living (3 RCTs), SMD, 0.51, 95%CIs, -0.08 to 1.10, P = 0.09, I^2 ^= 62%	...found limited effectiveness of moxibustion as an adjunct to standard care...	+

Lee (2010) [13]	Pain conditions	4 RCTs	Poor	7	Osteoarthritis: Moxa vs. drug (2 RCTs), RR, 1.11, 95%CIs, 1.02 to 1.21, P = 0.02, I^2 ^= 3%	...limited evidence ... in the management of osteoarthritis of the knee and other pain condition	+

Lee (2010) [14]	Constipation	3 RCTs	Poor	3	None	... evidence is scarce and insufficient to suggest...	**+/-**

Kim (2010) [15]	Hypertension	4 RCTs	Poor	5	None	There is insufficient...	**+/-**

^† ^This was relied on the original authors' judgment;* Overview quality assessment questionnaire (OQAQ). The overall score is from 1 to 7. OQAQ ≤ 3: having extensive or major flaws; 5 ≤ OQAQ: having minor or minimal flaws. +: overall positive; -: fail to show effectiveness; +/-: unclear. SR: systematic review; RCT: randomized controlled trial; CCT: non-randomized controlled trial.

For one particular condition, breech presentation, three SRs were available [7-9]. Two showed clearly positive results [8,9], whereas the other cast doubt on the clinical relevance of the effect due to the small number of included trials [7]. For the studies regarding cancer (10), stroke (11) and pain (12), the conclusions were cautiously positive. Equivocal conclusions emerged from the SRs on ulcerative colitis [10], constipation [14] and hypertension [15]. One SR evaluated the effects of moxibustion on various conditions and failed to arrive at a clearly positive conclusion [3].

## Discussion

Our overview shows that several SRs on moxibustion have been published. The fact that most of them were recent indicates that the scientific interest in moxibustion is growing. Even though most of the reviews are of high quality [3,7-15], they are based on few clinical trials that were not well controlled. Several SRs have arrived at positive overall conclusions; however, some of the studies regarding breech presentation contradict each other [7-9]. This can be explained by the time difference amongst the three SRs. The more up-to-date SR includes four to seven more rigorous and positive studies than the first SR [8,9]. Thus, the sum of the best evidence to date seems to suggest that moxibustion is effective for breech presentation.

Positive conclusions were also reached for the treatment for nausea and vomiting in cancer patients [11], stroke rehabilitation [12] and pain conditions [13]. Unfortunately, these SRs were based mostly on poor quality primary studies. Thus, considerable uncertainty about the value of moxibustion for these indications persists.

Three SRs relating to ulcerative colitis [10], constipation [14] and hypertension [15] were of poor quality, and all relied on a small number of flawed studies. It therefore seems fair to say that the value of moxibustion is not well-documented for any of these conditions.

In essence, this means that the effectiveness of moxibustion is currently not well-documented for several conditions, which is in sharp contrast to the many claims made by the proponents of this therapeutic modality, including those practicing traditional Chinese medicine or complementary and alternative medicine.

SRs on moxibustion have stated their studies to be limited by the often poor quality of the primary data. Our analysis confirms this view. Many of the primary moxibustion trials originate from China (data not shown); Vickers et al. demonstrated that virtually 100% of Chinese acupuncture trials are positive [16], which seems to be equally applied to moxibustion, an acupuncture-like intervention. This casts considerable doubt on the reliability of these studies. Collectively, these facts limit the conclusiveness of SRs on moxibustion, thereby leaving a level of uncertainty. SRs have been criticised for being often unable to provide specific guidance. Yet, even if uncertainty prevails, SRs have the important function of mapping areas of doubt. Thus, as pointed out above, our overview highlights areas of research in which investment in further clinical trials would be fruitful.

Thus our overview of SRs suggests that future moxibusition-research should consider all necessary measure to minimize bias including development of possible sham or placebo moxibustion. We recommend to follow the CONSORT guidelines when designing clinical trials of moxibustion [17]. Similarly, SRs of moxibustion should abide by the PRISMA guidelines to reduce the risk of bias [18].

Our overview has several important limitations. Even though our search strategy was thorough, we cannot completely exclude the notion that relevant articles were missed. By evaluating systematic reviews rather than clinical trials, important details of the primary studies may have been lost. Most importantly, the poor quality of the primary data and the systematic reviews is regrettable. Collectively, these limitations limit the conclusiveness of our findings.

In conclusion, this overview of SRs suggests that moxibustion is effective for correcting breech presentation, whereas for other conditions, the evidence does not reach a firm conclusion because of several limitations. All SRs are, however, based on studies with a high risk of bias. Therefore, considerable uncertainty remains about the therapeutic value of moxibustion.

## Competing interests

The authors declare that they have no competing interests.

## Authors' contributions

MSL, JWK and EE designed the review, performed searches, appraised and selected trials, extracted data, contacted authors for additional data, carried out analyses and interpretations of the data, and drafted this report. EE reviewed and critiqued the review protocol and this report and assisted in designing the review. All authors read and approved the final manuscript.

## References

[B1] World Health Organization Western Pacific Region WHO International Standard Terminologies on Traditional Medicine in the Western Pacific Region http://www.wpro.who.int/publications/PUB_9789290612487.htm Accessed at 21 Oct, 2010

[B2] Korean Acupuncture & Moxibustion Society Acupuncture and Moxibustion 2008 Seoul: Jibmundang

[B3] Kim S-Y Chae Y Lee SM Lee H Park H-J The effectiveness of moxibustion: an overview during 10 years eCAM 2009 nep163 19825873 10.1093/ecam/nep163 PMC3136359

[B4] Li GR Li QY Clinical Moxibustion Therapy 2007 2 Beijing: People's Medical Publishing House

[B5] Zhao JP Wang YP Acupuncture and Moxibustion 2007 Beijing: People's Medical Publishing House

[B6] Oxman AD Guyatt GH Validation of an index of the quality of review articles J Clin Epidemiol 1991 44 11 1271 1278 10.1016/0895-4356(91)90160-B 1834807

[B7] Coyle ME Smith CA Peat B Cephalic version by moxibustion for breech presentation Cochrane Database Syst Rev 2005 2 CD003928 15846688 10.1002/14651858.CD003928.pub2

[B8] Li X Hu J Wang X Zhang H Liu J Moxibustion and other acupuncture point stimulation methods to treat breech presentation: a systematic review of clinical trials Chin Med 2009 4 4 10.1186/1749-8546-4-4 19245719 PMC2663768

[B9] Vas J Aranda JM Nishishinya B Mendez C Martin MA Pons J Liu JP Wang CY Perea-Milla E Correction of nonvertex presentation with moxibustion: a systematic review and metaanalysis Am J Obstet Gynecol 2009 201 3 241 259 10.1016/j.ajog.2008.12.026 19733275

[B10] Lee DH Kim JI Lee MS Choi TY Choi SM Ernst E Moxibustion for ulcerative colitis: a systematic review and meta-analysis BMC Gastroenterol 2010 10 36 10.1186/1471-230X-10-36 20374658 PMC2864201

[B11] Lee MS Choi TY Park JE Lee SS Ernst E Moxibustion for cancer care: a systematic review and meta-analysis BMC Cancer 2010 10 130 10.1186/1471-2407-10-130 20374659 PMC2873382

[B12] Lee MS Shin BC Kim JI Han CH Ernst E Moxibustion for stroke rehabilitation: systematic review Stroke 2010 41 4 817 820 10.1161/STROKEAHA.109.566851 20150551

[B13] Lee MS Choi TY Kang JW Lee BJ Ernst E Moxibustion for treating pain: a systematic review Am J Chin Med 2010 38 5 829 838 10.1142/S0192415X10008275 20821815

[B14] Lee MS Choi TY Park JE Ernst E Effects of moxibustion for constipation: a systematic review of randomized controlled trials Chin Med 2010 5 28 10.1186/1749-8546-5-28 20687948 PMC2922210

[B15] Kim JI Choi JY Lee H Lee MS Ernst E Moxibustion for hypertension: a systematic review BMC Cardiovasc Disord 2010 10 1 33 10.1186/1471-2261-10-33 20602794 PMC2912786

[B16] Vickers A Goyal N Harland R Rees R Do certain countries produce only positive results? A systematic review of controlled trials Control Clin Trials 1998 19 2 159 166 10.1016/S0197-2456(97)00150-5 9551280

[B17] Schulz K Altman D Moher D Consort Group CONSORT 2010 Statement: updated guidelines for reporting parallel group randomised trials BMC Medicine 2010 8 1 18 10.1186/1741-7015-8-18 20334633 PMC2860339

[B18] PRISMA Preferred Reporting Items of Systematic reviews and Meta-Analyses (PRISMA) Statement http://www.prisma-statement.org/index.htm Accessed at 21 Oct, 2010

